# ALK-Targeted Therapy: Resistance Mechanisms and Emerging Precision Strategies

**DOI:** 10.3390/cimb47120996

**Published:** 2025-11-27

**Authors:** Ya-Kun Zhang, Jian-Bo Tong, Mu-Xuan Luo, Zhi-Peng Qin, Rong Wang

**Affiliations:** 1 College of Chemistry and Chemical Engineering, Shaanxi University of Science and Technology, Xi’an 710021, China; 2 State Key Laboratory of Advanced Processing and Recycling of Non-ferrous Metals, Lanzhou University of Technology, Lanzhou 730050, China

**Keywords:** anaplastic lymphoma kinase, resistance mechanisms, inhibitors, targeted therapy, precision medicine

## Abstract

Anaplastic lymphoma kinase (ALK), a member of the receptor tyrosine kinase family, plays a central oncogenic role in the initiation and progression of diverse malignancies. Aberrant ALK activation generally results from structural alterations or dysregulated expression, leading to persistent activation of downstream signaling pathways that drive tumor cell proliferation, survival, and metastasis. *ALK* gene abnormalities predominantly encompass fusions, point mutations, and amplifications, with EML4-ALK-positive non–small cell lung cancer representing a canonical example. The advent of ALK-targeted inhibitors has constituted a major therapeutic milestone for ALK-positive tumors. From first-generation Crizotinib to third-generation Lorlatinib, successive agents have been refined for target selectivity, central nervous system penetration, and coverage of resistance-associated mutations, substantially improving patient survival and intracranial disease control. Nonetheless, the emergence of acquired resistance remains an overarching challenge, mediated by secondary kinase domain mutations, activation of bypass signaling pathways, and tumor phenotypic transformation. This review presents an integrative synthesis of ALK-targeted therapeutic developments, elucidates underlying resistance mechanisms, and surveys emerging strategies, providing a comprehensive perspective on current advances and future directions in precision management of ALK-driven malignancies.

## 1. Introduction

Anaplastic lymphoma kinase (ALK), a member of the receptor tyrosine kinase (RTK) family [1], has garnered considerable attention owing to its pivotal oncogenic driver role across multiple malignancies [2]. Structurally, ALK comprises an extracellular ligand-binding domain, a transmembrane segment, and an intracellular catalytic kinase domain, which collectively regulate essential cellular processes such as proliferation, differentiation, and survival [3]. The elucidation of ALK’s structural and dynamic features through high-resolution crystallography and molecular modeling has provided a robust framework for structure-based targeted therapeutic design, while also offering insight into how structural aberrations contribute to its oncogenic activation.

The oncogenic potential of *ALK* is predominantly driven by genetic alterations, including fusion rearrangements, most notably the EML4-ALK fusion in non-small cell lung cancer, activating point mutations, and gene amplification [4]. These alterations induce conformational changes that result in constitutive kinase activation, thereby driving malignant transformation and uncontrolled proliferation [5]. The tumorigenic effects of ALK are critically mediated through sustained dysregulation of key signaling pathways, including the RAS/mitogen-activated protein kinase (RAS/MAPK), phosphatidylinositol 3-kinase/protein kinase B (PI3K/AKT), and Janus kinase/signal transducer and activator of transcription (JAK/STAT) signaling axes [6,7]. These pathways act synergistically to perturb the cell cycle, inhibit apoptosis, and remodel the tumor immune microenvironment, ultimately promoting aggressive tumor progression and metastasis.

Tyrosine kinase inhibitors targeting ALK structural aberrations (ALK-TKIs) have emerged as a cornerstone in the treatment of ALK-positive malignancies, particularly non-small cell lung cancer [8], evolving from first-generation Crizotinib to third-generation Lorlatinib [9,10,11,12]. Next-generation inhibitors provide broader coverage of resistance-associated mutations, such as L1196M and G1202R, and exhibit markedly enhanced central nervous system penetration, thereby improving progression-free survival [13]. Nevertheless, the development of acquired resistance is almost inevitable, underpinned by highly heterogeneous mechanisms, including secondary kinase domain mutations, activation of bypass signaling pathways (MET amplification, EGFR upregulation), and tumor phenotypic transformations such as epithelial–mesenchymal transition (EMT) [14]. These mechanisms collectively establish a complex resistance network that substantially diminishes therapeutic efficacy.

Against this backdrop, an in-depth understanding of the molecular bases of ALK inhibitor resistance and their dynamic evolutionary trajectories is indispensable for the rational design of next-generation inhibitors and the optimization of combination strategies. This review aims to systematically integrate the molecular architecture and functional characteristics of ALK, summarize the latest advances in ALK-targeted therapies, elucidate the molecular mechanisms underlying drug resistance, and outline emerging strategies, thereby providing a comprehensive scholarly reference and theoretical framework for the precision management of ALK-driven malignancies. This review retrieved relevant literature on “ALK inhibitors,” “resistance mechanisms,” and “combination strategies” from PubMed and Web of Science (2015–2025), with manual screening and cross-referencing to ensure the studies included are representative and up-to-date.

## 2. Molecular and Functional Basis of ALK

### 2.1. Genomic and Structural Characteristics of ALK

**Figure 1:**
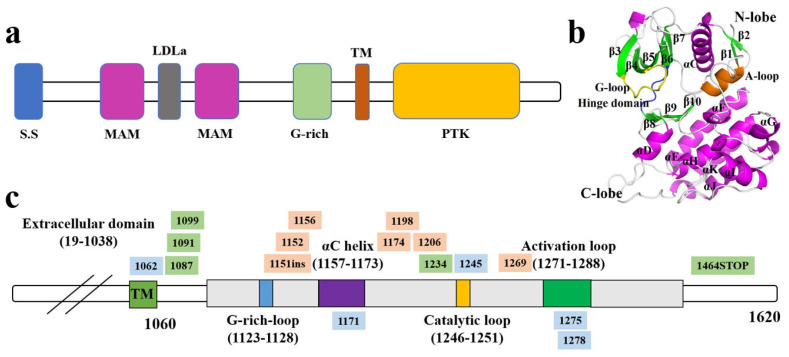
Schematic representation of ALK structure and kinase domain; (**a**): Domain architecture of ALK protein; (**b**): Three-dimensional structure of the ALK kinase domain; (**c**): Structural-functional mapping and mutational landscape of ALK.

ALK is a transmembrane receptor tyrosine kinase within the insulin receptor tyrosine kinase family. The *ALK* gene is located on the short arm of human chromosome 2 (2p23) and comprises 29 exons, encoding a polypeptide of 1620 amino acids with a theoretical molecular weight of approximately 180 kDa [15]. Structurally, ALK is organized from the N-terminus to the C-terminus into a signal peptide, two MAM domains, an LDL-A-like domain, a G-rich region, a single-pass transmembrane helix, and a C-terminal intracellular protein tyrosine kinase (PTK) domain [16], forming a canonical receptor tyrosine kinase architecture (Figure 1a). The extracellular segment primarily mediates ligand recognition and conformational regulation, the transmembrane domain anchors the receptor to the plasma membrane, and the intracellular kinase domain constitutes the catalytic core responsible for downstream signaling [17].

The three-dimensional structure of the ALK kinase domain adopts a bilobed configuration, comprising an N-lobe and a C-lobe, and incorporates several highly conserved motifs critical for catalytic function, including the G-loop, hinge region, αC helix, catalytic loop, and activation loop (Figure 1b). These structural elements are essential for maintaining conformational stability, facilitating ATP and substrate binding, and regulating kinase activation, thereby representing central targets for mechanistic studies and inhibitor design.

Mapping of the full-length primary sequence further delineates the functional regions and highlights mutational hotspots (Figure 1c). The G-rich loop (residues 1123–1128), αC helix (1157–1173), catalytic loop (1246–1251), and activation loop (1271–1288) constitute the kinase’s functional core, within which multiple acquired mutations, including 1151ins, activating mutations at 1269, and truncating 1464STOP are densely clustered [18]. These alterations frequently enhance kinase activity or perturb conformational stability, resulting in sustained activation of downstream signaling cascades, including PI3K-AKT, RAS-MAPK, and JAK-STAT pathways [19]. Such dysregulation markedly promotes tumor cell proliferation and survival and plays a critical role in mediating resistance to ALK-targeted therapies.

### 2.2. Physiological Function and Expression Profile of ALK

Under normal physiological conditions, ALK exhibits highly restricted spatiotemporal expression, predominantly confined to specific regions of the embryonic and perinatal nervous system, including neural crest-derived sympathetic ganglia, dorsal root ganglia, and brainstem [20]. As a canonical receptor tyrosine kinase, ALK transduces signals via its conserved transmembrane and intracellular kinase domains to orchestrate critical neurodevelopmental processes, such as neuronal lineage specification, axon guidance, synaptogenesis, and neural circuit formation [21]. Mechanistic studies and animal models indicate that ALK’s functions in neural development are non-redundant and highly specific, regulating neuronal migration, survival, and synaptic plasticity. Following completion of development, ALK expression is markedly downregulated in adult tissues, with transcriptomic and proteomic analyses (GTEx, Human Protein Atlas) revealing minimal or negligible expression across most tissues and only limited presence in select cell subpopulations [22]. This restricted adult expression underlies both its physiological specificity and its therapeutic relevance: the absence of ALK in most normal tissues renders it an attractive tumor-specific target, allowing for effective antitumor intervention while potentially minimizing on-target toxicity, thereby enhancing the feasibility and safety profile of ALK-targeted therapies.

### 2.3. Mechanisms of Activation and Oncogenic Signaling

Although ALK exhibits a restricted expression profile and highly specific functional roles under normal physiological conditions, its aberrant activation has been widely recognized as a critical oncogenic driver in multiple malignancies. Constitutive ALK kinase activation arises primarily through three mechanisms: gene rearrangements generating fusion proteins, activating point mutations, and gene amplification [23,24]. Canonical ALK fusion proteins, such as EML4-ALK, undergo dimerization mediated by the N-terminal fusion partner, stabilizing the C-terminal tyrosine kinase domain and persistently activating downstream signaling axes, including RAS-MAPK, PI3K-AKT, and JAK-STAT, thereby promoting tumor cell proliferation, survival, and migration [25]. Point mutations are commonly observed as primary oncogenic events in neuroblastoma (F1174L, R1275Q) or as secondary resistance mutations emerging following ALK inhibitor therapy (G1202R, L1196M) [26,27], which reduce inhibitor binding efficiency via enhanced ATP affinity or conformational alterations [28]. Although reports of ALK gene amplification are relatively infrequent, the resultant upregulation of protein expression can similarly augment signaling activity and contribute to tumorigenesis. In diverse tumor subtypes, these activating mechanisms may coexist in a synergistic or sequential manner, providing a fundamental basis for malignant clonal evolution and disease progression.

### 2.4. Associated Signaling Pathways

**Figure 2:**
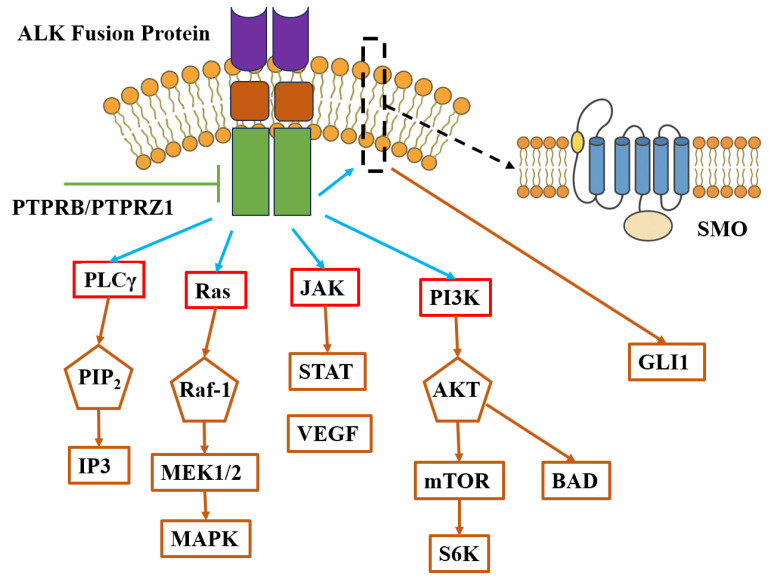
ALK-associated signaling pathways.

ALK fusion proteins, through constitutive activation of their tyrosine kinase domains, drive persistent activation of multiple downstream signaling pathways, including canonical oncogenic axes such as Ras/MAPK, PI3K/AKT/mTOR, JAK/STAT, and PLCγ [29], thereby coordinately regulating critical biological processes such as cell cycle progression, survival and metabolic reprogramming, immune evasion, and adhesion and migration. Specifically, the Ras/MAPK pathway primarily mediates cell proliferation and differentiation [30], the PI3K/AKT/mTOR axis regulates anti-apoptotic responses and nutrient stress adaptation, and the JAK/STAT pathway, particularly STAT3, sustains proliferative signaling in various ALK-positive tumors [31]. The PLCγ pathway contributes to cytoskeletal remodeling and motility regulation via calcium signaling and PKC activation [32]. Beyond canonical signaling axes, ALK may engage non-canonical mechanisms, such as aberrant activation of the Hedgehog/SMO-GLI axis [33], which has been observed in preclinical tumor models and may contribute to tumor stemness and resistance. However, evidence remains limited and lacks systematic clinical validation. Notably, ALK signaling activity is subject to negative regulation by protein tyrosine phosphatases PTPRB and PTPRZ1, which suppress kinase activity through dephosphorylation. However, in multiple ALK fusion-positive tumors, these negative regulators are frequently downregulated or functionally impaired, potentially further promoting sustained ALK signaling and maintaining the phenotypic stability of malignant clones (Figure 2).

## 3. Role of Aberrant ALK in Diverse Malignancies

**Figure 3:**
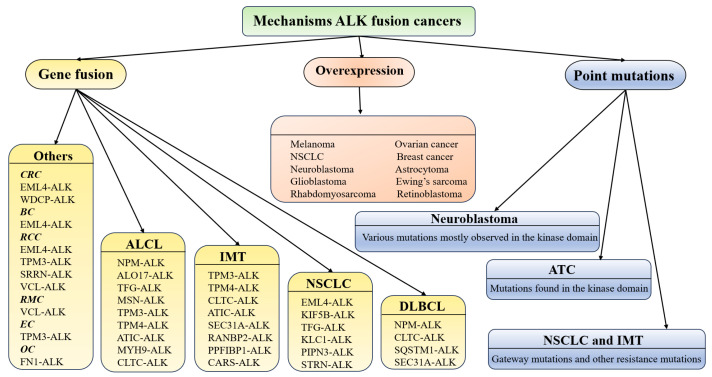
Mechanisms of ALK activation in fusion-driven cancers.

Aberrant activation of ALK serves as a pivotal oncogenic driver in a wide range of solid tumors and hematological malignancies, broadly contributing to tumor initiation, progression, and invasiveness (Figure 3). In non-small cell lung cancer (NSCLC), ALK fusion proteins, exemplified by EML4-ALK, promote tumor cell proliferation, survival, and migration through persistent activation of downstream oncogenic signaling pathways, representing a key molecular subtype and a primary target for precision therapy [34]. In anaplastic large cell lymphoma (ALCL), ALK gene rearrangements frequently lead to fusion protein expression, driving malignant transformation and expansion of lymphoid cells [35]. Activating point mutations and gene amplification of ALK in neuroblastoma have also been implicated in enhanced tumor aggressiveness and therapeutic resistance. Moreover, aberrant ALK activation has been reported in diverse malignancies, including thyroid cancer, breast cancer, and certain soft tissue sarcomas, highlighting the broad spectrum of its oncogenic impact [36]. Through sustained signaling via multiple pathways, ALK regulates critical biological processes such as cell cycle progression, apoptosis evasion, and cellular migration, underscoring its significance as a molecular target for precision oncology.

### 3.1. NSCLC

In NSCLC, ALK aberrations most frequently manifest as gene rearrangements, with the EML4-ALK fusion being the prototypical example, detected in approximately 3–7% of lung adenocarcinomas [37]. This fusion results from an inversion on the short arm of chromosome 2, generating a chimeric protein that retains the intracellular kinase domain of ALK and achieves conformational stabilization through the dimerization domain of EML4, leading to constitutive kinase activation [38]. The oncogenic ALK fusion continuously engages multiple downstream signaling pathways, including PI3K-AKT, RAS-MAPK, and JAK-STAT, thereby promoting tumor cell proliferation, migration, and evasion of apoptosis [39,40]. Clinically, ALK-rearranged NSCLC is more frequently observed in younger patients with minimal or no smoking history and is predominantly associated with mucinous or solid-type adenocarcinomas [41]. Given its well-established role as a driver of tumorigenesis, ALK rearrangement has emerged as a key molecular biomarker and therapeutic target in NSCLC.

### 3.2. ALCL

In ALCL, ALK aberrations predominantly arise from the chromosomal translocation t(2;5) (p23;q35), giving rise to the canonical NPM1-ALK fusion protein [42]. This chimeric protein retains the intracellular kinase domain of ALK and undergoes constitutive activation via NPM1-mediated dimerization, leading to persistent engagement of key oncogenic signaling pathways, including STAT3, PI3K-AKT, and RAS-MAPK. Among these, sustained activation of the STAT3 axis has been implicated as a central mechanism supporting tumor cell proliferation, immune evasion, and resistance to apoptosis [43]. Furthermore, NPM1-ALK may promote tumor invasiveness through chromatin remodeling and transcriptional reprogramming. Clinically, ALK-positive ALCL predominantly affects children and young adults and exhibits a characteristic immunophenotype (CD30-positive, ALK-positive) [44,45]. These cases generally display favorable responses to conventional chemotherapy and ALK-targeted inhibitors, with overall prognosis superior to ALK-negative counterparts. Collectively, NPM1-ALK represents both a pivotal molecular diagnostic marker and a critical therapeutic target, underscoring its significance in the pathobiology and clinical management of ALCL.

### 3.3. Others

In neuroblastoma, ALK aberrations are predominantly point mutations within the kinase domain, with R1275Q and F1174L representing the most frequent variants [46]. These oncogenic mutations enhance the conformational stability and autophosphorylation of ALK, resulting in constitutive activation of key downstream signaling pathways, including RAS-MAPK, PI3K-AKT, and JAK-STAT, thereby driving malignant transformation of neural crest–derived cells [47]. Notably, ALK mutations often co-occur with MYCN amplification, and this co-alteration appears to synergistically facilitate tumor initiation and progression. Certain mutation subtypes exhibit intrinsic resistance to first-generation ALK inhibitors, such as crizotinib, which has spurred the development and clinical evaluation of more potent agents targeting mutant ALK, including lorlatinib.

In inflammatory myofibroblastic tumors (IMTs), ALK aberrations primarily involve gene rearrangements, with ALK fusions detected in approximately 50–60% of cases [48]. Fusion partners are diverse, including TPM3, TPM4, CLTC, and ATIC, and the resultant chimeric proteins mediate constitutive ALK activation, promoting aberrant cellular proliferation and remodeling of the inflammatory tumor microenvironment [42]. ALK-positive IMTs predominantly arise in children and young adults and are histologically characterized by myofibroblastic proliferation accompanied by dense inflammatory infiltrates, with immunohistochemistry demonstrating cytoplasmic and nuclear ALK expression [22]. In unresectable or recurrent cases, ALK-targeted therapy, particularly with crizotinib [48], has demonstrated significant clinical efficacy, thereby establishing a rationale for precision therapy in this patient population.

## 4. Evolution of ALK Inhibitors

**Figure 4:**
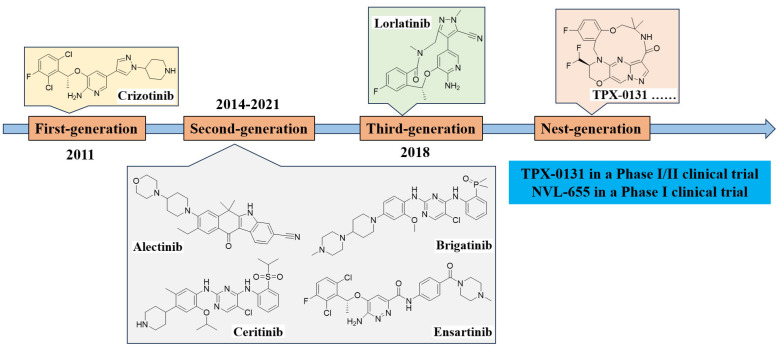
Chronological development of ALK inhibitors.

With increasing insights into the molecular mechanisms underlying ALK-driven malignancies, the development of ALK inhibitors has progressed from first-generation to successive next-generation agents. This evolution has addressed key clinical challenges, including acquired resistance and central nervous system metastases, thereby markedly enhancing the therapeutic prospects for patients harboring ALK-positive tumors (Figure 4).

### 4.1. First-Generation ALK Inhibitor: Crizotinib

Crizotinib, the first FDA-approved ALK tyrosine kinase inhibitor, received regulatory approval in 2011 and rapidly became the preferred targeted therapy for patients with ALK-positive NSCLC [49]. Crizotinib exerts its antitumor effect by competitively binding to the ATP-binding pocket of the ALK kinase domain, thereby inhibiting kinase activity and suppressing aberrant activation of critical oncogenic signaling pathways, including PI3K-AKT and RAS-MAPK [11]. In addition, Crizotinib also inhibits ROS1 and MET kinases, reflecting its multi-target profile [50,51]. Clinical studies have demonstrated that Crizotinib significantly improves objective response rates (ORRs) and progression-free survival (PFS) compared with conventional chemotherapy, with a favorable safety profile [52]. Nevertheless, with prolonged treatment, acquired resistance frequently emerges, primarily mediated by secondary mutations in the ALK kinase domain (the L1196M “gatekeeper” mutation), activation of bypass signaling pathways, and limited central nervous system penetration, all of which constrain its long-term efficacy.

### 4.2. Second-Generation ALK Inhibitors: Ceritinib, Alectinib, and Brigatinib

To address resistance to Crizotinib, second-generation ALK inhibitors were developed to enhance potency against certain resistance-associated mutations and improve central nervous system (CNS) penetration. Representative agents, including Ceritinib, Alectinib, and Brigatinib, exhibit increased affinity and selectivity for the ALK ATP-binding pocket [53], showing improved activity against several resistant mutations such as L1196M and F1174C [54]. However, mutations such as G1202R are poorly inhibited by second-generation agents and are primarily targeted by third-generation inhibitors, such as Lorlatinib. Ceritinib was among the earliest approved second-generation inhibitors and demonstrated prolonged PFS and OS in Crizotinib-resistant patients. Alectinib, notable for its favorable safety profile and CNS efficacy, has become a standard first-line therapy, reducing the incidence of CNS progression [12]. Brigatinib, with its unique molecular design, shows rapid onset and good tolerability, providing robust activity against difficult-to-treat mutations [55], including some resistant variants [56]. Overall, second-generation ALK inhibitors significantly expand treatment options and overcome multiple resistance mechanisms associated with first-generation agents, while certain mutations still require third-generation therapies [57].

### 4.3. Third-Generation ALK Inhibitor: Lorlatinib

Lorlatinib, a third-generation ALK inhibitor, was specifically developed to overcome a broad spectrum of resistance mutations that are refractory to earlier-generation agents [58], particularly the challenging G1202R “gatekeeper” mutation [59], demonstrating potent inhibitory activity [60]. Its molecular design is optimized to accommodate resistant conformations while exhibiting excellent blood–brain barrier penetration, substantially enhancing therapeutic efficacy against CNS metastases [61]. Multiple clinical studies have confirmed that Lorlatinib achieves favorable ORR and PFS even in patients who have experienced treatment failure with first and second-generation ALK inhibitors [10,62]. Although associated with certain central nervous system-related adverse effects, Lorlatinib is generally well tolerated and significantly expands therapeutic options for patients with ALK-positive NSCLC, representing a frontier in current ALK-targeted therapy.

### 4.4. Next-Generation ALK Candidates and Therapeutic Strategies

With the widespread clinical application of ALK inhibitors, resistance mechanisms have become increasingly diverse and complex, driving the development of next-generation agents and integrated therapeutic strategies. Current efforts are focused on designing ALK inhibitors that provide broader coverage of resistance-associated mutations while exhibiting enhanced CNS penetration, thereby addressing challenges such as compound mutations, bypass signaling activation, and tumor heterogeneity [63]. Emerging candidates, including TPX-0131 and NVL-655 [64,65], have demonstrated high affinity for difficult-to-treat mutations, particularly G1202R and compound alterations, and exhibit superior control of CNS metastases in preclinical models. Their unique molecular designs, such as macrocyclic or flexible scaffolds, optimize binding to the ATP-binding pocket and improve pharmacokinetic properties. Some of these candidates have advanced to early-phase clinical trials, providing promising avenues for future therapy. Overall, next-generation ALK inhibitors, through structural optimization and enhanced pharmacologic profiles, hold the potential not only to overcome existing resistance barriers but also to enable precise modulation of tumor biology, ultimately improving therapeutic efficacy and tolerability.

## 5. Resistance Mechanisms to ALK Inhibitors

**Figure 5:**
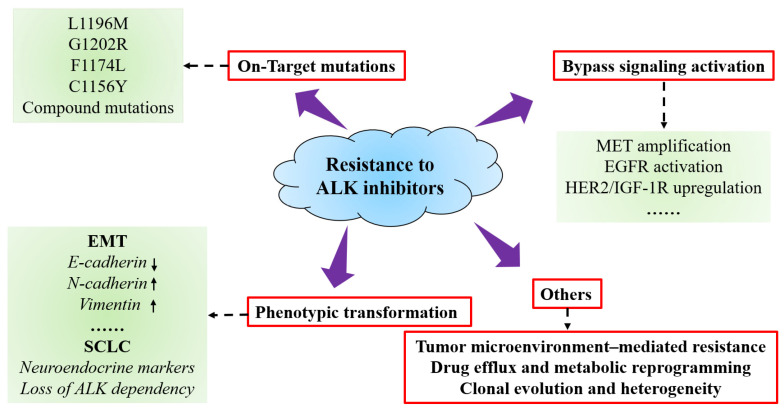
Mechanisms of resistance to ALK inhibitors.

The mechanisms underlying resistance to ALK inhibitors are complex and multifaceted. Traditionally, resistance has been categorized into three major types: on-target mutations, bypass pathway activation, and phenotypic transformation (Figure 5) [66]. However, accumulating evidence indicates that these classical mechanisms alone do not fully account for the spectrum of clinical resistance [8,67]. In addition to these canonical pathways, tumor microenvironment–mediated protective effects, drug efflux, and metabolic reprogramming, as well as clonal evolution and intratumoral heterogeneity, have been shown to play critical roles in the development of resistance [23,68]. These mechanisms are highly interrelated, forming a dynamic and multilayered resistance network that collectively enables tumor cells to evade targeted therapy, posing a significant challenge to precision oncology in the clinical setting.

### 5.1. On-Target Mutations

On-target mutations represent a pivotal mechanism of acquired resistance to ALK inhibitors, predominantly involving structural alterations of critical residues within the ALK tyrosine kinase domain. These modifications profoundly reshape the kinase conformation and the physicochemical landscape of the ATP-binding pocket, thereby attenuating inhibitor binding affinity and compromising therapeutic efficacy [69]. The L1196M mutation, a prototypical “gatekeeper” alteration, substitutes the residue at the entrance of the ATP-binding channel, generating steric hindrance that substantially impairs the engagement of first-generation inhibitors such as Crizotinib [70]. The G1202R mutation, located at the solvent-front region, introduces a bulky arginine side chain, creating severe steric clash and rendering most first- and second-generation ALK inhibitors largely ineffective; it remains one of the most formidable resistance mutations in the clinical setting [71]. Additional mutations, including F1174L and C1156Y, stabilize the kinase active conformation and increase ATP affinity, thereby sustaining enzymatic activity and reinforcing the resistant phenotype. Importantly, the emergence of compound mutations has become increasingly prevalent with the clinical use of sequential ALK inhibitors. Combinatorial alterations, such as G1202R + L1196M or G1202R + F1174C, act synergistically to amplify resistance and substantially limit the efficacy of available therapeutics. The structural diversity, adaptive potential, and dynamic evolution of on-target mutations not only exemplify tumor cell remodeling under selective pressure from targeted therapy but also provide a precise framework for the rational design of next-generation, broad-spectrum ALK inhibitors.

### 5.2. Bypass Signaling Activation

Beyond on-target mutations, activation of bypass signaling pathways constitutes a critical mechanism of resistance to ALK inhibitors [72]. Tumor cells circumvent ALK dependency by initiating or upregulating alternative receptor tyrosine kinases (RTKs) and their downstream signaling cascades, thereby sustaining cellular proliferation and survival [54]. Common bypass activations include MET gene amplification, EGFR activation, and upregulation of other RTKs such as HER2 and IGF-1R. These alternative pathways functionally compensate for ALK signaling by activating key downstream effectors, including PI3K-AKT, RAS-MAPK, and JAK-STAT [73]. Among them, MET amplification is frequently observed across various ALK inhibitor resistance models and confers enhanced migratory, invasive, and anti-apoptotic phenotypes, representing a prototypical ALK-independent escape mechanism. In addition to RTK-mediated bypass, feedback signals from cytokines and immune cells within the tumor microenvironment can further promote activation of alternative pathways, enhancing tumor cell adaptability to targeted therapy. Notably, these bypass alterations may be present prior to treatment (de novo), emerge under selective pressure from ALK TKIs (emergent), or result from a combination of both mechanisms. The pronounced heterogeneity and plasticity of bypass signaling significantly limit the efficacy of single-agent inhibitors, underscoring the urgent need for multi-pathway combination strategies in the clinical management of resistance mediated by alternative signaling circuits.

### 5.3. Phenotypic Transformation

Phenotypic transformation represents a critical mechanism of resistance to ALK inhibitors, characterized by tumor cells undergoing lineage reprogramming to shift from an ALK-dependent phenotype to alternative cellular states, thereby evading the selective pressure imposed by targeted therapy [61]. Typical forms of phenotypic transformation include EMT and SCLC-like transdifferentiation. During EMT, tumor cells acquire enhanced migratory, invasive, and anti-apoptotic capabilities, accompanied by cytoskeletal remodeling and altered expression of hallmark molecular markers, such as downregulation of E-cadherin and upregulation of N-cadherin and vimentin, resulting in markedly reduced sensitivity to ALK inhibitors [74]. In addition, a subset of ALK-positive NSCLC patients may undergo SCLC-like transformation during therapy, exhibiting neuroendocrine differentiation and loss of ALK dependency, which leads to a precipitous decline in therapeutic efficacy [75]. This process is often accompanied by complex transcriptional reprogramming and epigenetic remodeling, with inactivation of tumor suppressors such as TP53 and RB1 playing a pivotal role. Phenotypic transformation not only reflects the remarkable plasticity and adaptive capacity of tumor cells under therapeutic pressure but also highlights the limitations of ALK-targeted monotherapy, supporting the rationale for the development of combination strategies incorporating phenotype-reversing agents or other adjunctive therapeutic modalities.

### 5.4. Other Mechanisms

Beyond classical ALK on-target mutations, tumor resistance involves a spectrum of “noncanonical” mechanisms. The tumor microenvironment contributes to therapeutic evasion by establishing a protective niche through fibroblast-derived factors, infiltration of immunosuppressive cells, and extracellular matrix remodeling, thereby attenuating the efficacy of ALK inhibitors [76]. Concurrently, drug efflux and metabolic reprogramming represent additional resistance pathways: upregulation of ABC transporters coupled with enhanced glycolysis and lipid biosynthesis not only reduces intracellular drug concentrations but also provides supplementary energy to sustain proliferation and survival. Moreover, clonal evolution and tumor heterogeneity enable distinct cellular clones to alternately dominate under selective pressure, exhibiting spatiotemporal variability and dynamic evolution of resistance mechanisms. The pronounced heterogeneity and plasticity of these processes significantly limit the efficacy of single-target therapies, underscoring the need for multidimensional approaches including structure-guided inhibitor redesign, pathway-targeted combination regimens, and biomarker-informed adaptive therapy guided by longitudinal molecular profiling. Integration of single-cell sequencing, circulating tumor DNA (ctDNA) monitoring, and spatial multi-omics analyses may further enable early detection of resistance evolution and guide precision treatment adaptation.

## 6. Strategies to Overcome Resistance

**Figure 6:**
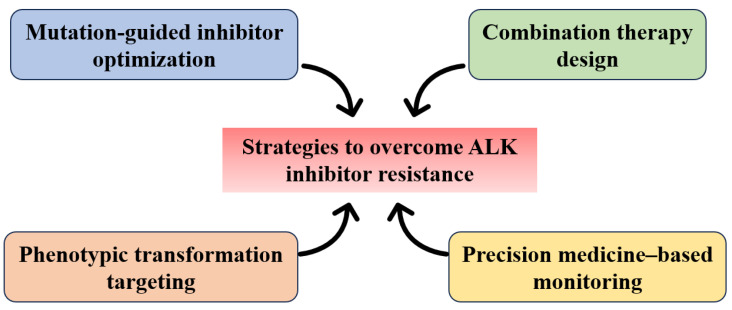
Strategies to overcome resistance to ALK-targeted therapy.

Although ALK inhibitors have demonstrated remarkable clinical efficacy in the treatment of ALK fusion–positive tumors, substantially prolonging patient survival, the widespread emergence of secondary resistance continues to limit their long-term effectiveness. Given the high heterogeneity and mechanistic complexity of resistance to ALK-targeted therapy, current clinical and preclinical research primarily focuses on four areas: optimization of inhibitors based on resistance mutation profiles, rational design of combination therapies, precise interventions targeting phenotypic transformation, and dynamic molecular monitoring coupled with individualized treatment adjustments guided by precision medicine frameworks (Figure 6) [77]. The integrated application of these strategies aims to enhance the overall efficacy of ALK-targeted therapy and delay the onset of resistance, thereby providing a more robust theoretical and practical foundation for sustained control of ALK-positive malignancies.

### 6.1. Resistance Mutation-Guided ALK Inhibitor Optimization

Resistance to ALK inhibitors is largely driven by diverse secondary mutations within the kinase domain, which alter the ATP-binding pocket and reduce inhibitor affinity, ultimately compromising therapeutic efficacy. To overcome such structural resistance, drug development is increasingly shifting from empirically driven approaches toward precision-guided optimization based on comprehensive mutation profiling. First-generation ALK inhibitors, such as Crizotinib, exhibit limited efficacy against several resistance mutations (L1196M, G1269A), driving the development of successive second- and third-generation inhibitors. Representative molecules like Lorlatinib enhance molecular rigidity and polar interactions, thereby significantly improving inhibition of key mutations, including G1202R and L1196M, and demonstrating broad-spectrum anti-mutation activity. Emerging candidates, such as TPX-0131 and NVL-655, further focus on compound mutation scenarios, employing highly flexible scaffolds and conformational tuning strategies to expand coverage of heterogeneous resistant configurations. Rational optimization guided by resistance mutation spectra not only broadens the therapeutic applicability of inhibitors but also signifies a paradigm shift in ALK-targeted therapy from passive response to proactive prediction and precision intervention.

### 6.2. Combination Therapeutic Strategies

Given the limited efficacy of ALK inhibitors as monotherapy in overcoming diverse resistance mechanisms, combination therapeutic strategies have emerged as a critical approach to delay resistance onset and enhance treatment outcomes. Such combination regimens are predicated on a deep understanding of the underlying molecular mechanisms of resistance, enabling multi-layered, synergistic inhibition of distinct resistance pathways. For bypass signaling–mediated resistance, including upregulation of MET, EGFR, and other pathways, co-administration of ALK inhibitors with corresponding targeted agents (Crizotinib plus Osimertinib, Lorlatinib plus Capmatinib) has demonstrated promising antitumor activity in both clinical and preclinical studies [78,79]. For resistance driven by phenotypic transformation, the inclusion of TGF-β pathway inhibitors or HDAC inhibitors can reverse EMT phenotypes and restore sensitivity to ALK inhibition [80]. Moreover, the combination of ALK inhibitors with immune checkpoint inhibitors has demonstrated potential synergistic antitumor activity; however, the inherent complexity of the immune microenvironment and the distinct toxicity risks associated with TKI–ICI combinations necessitate careful patient selection and thoughtful optimization of treatment sequencing [81]. Notably, the addition of ICIs on a TKI backbone markedly increases the incidence of immune-related adverse events such as immune-mediated hepatitis and interstitial pneumonitis, which may further compound the intrinsic hepatic, pulmonary, and hematologic toxicities of ALK inhibitors, thereby constraining the clinical feasibility of such regimens [82,83]. Thus, although combination strategies offer enhanced depth and breadth of pathway suppression, their successful clinical implementation remains contingent upon meticulous toxicity management, assessment of patient tolerability, and the integration of molecularly informed precision-medicine frameworks to achieve an optimal balance between efficacy and safety.

### 6.3. Intervention Strategies Targeting Phenotypic Transformation

Phenotypic transformation represents a critical mechanism underlying resistance to ALK inhibitors, primarily manifested through EMT and SCLC-like conversion, enabling tumor cells to escape ALK-dependent signaling. Intervention strategies targeting this mechanism focus on multi-layered regulation of cell fate and transcriptional networks. EMT-associated resistance has been partially reversed in both in vitro and in vivo models through targeting TGF-β, Wnt/β-catenin pathways, and epigenetic modulators, thereby restoring tumor sensitivity to ALK inhibitors [84]. SCLC-like transformation, often accompanied by TP53 and RB1 inactivation [85], necessitates therapeutic approaches informed by small cell lung cancer paradigms, including chemotherapy, PARP inhibitors, and DLL3-targeted antibody drug conjugates [86,87]. Multi-modal longitudinal monitoring technologies, such as single-cell RNA sequencing and spatial transcriptomics, provide powerful tools to dynamically track transformation processes and intratumoral heterogeneity, supporting the design of precision intervention strategies [88]. Overall, phenotypic transformation–targeted interventions exemplify a shift from single-target inhibition toward integrated mechanistic modulation, serving as a crucial complement in overcoming resistance to ALK-targeted therapy.

### 6.4. Precision Medicine Guided Approaches

Precision medicine–guided approaches leverage high-throughput omics technologies, liquid biopsies, and dynamic molecular monitoring to enable real-time tracking and precise intervention during the development of resistance to ALK inhibitors [89]. Liquid biopsy techniques, such as circulating tumor DNA (ctDNA) analysis [90], sensitively detect resistance mutations and bypass pathway activations, providing molecularly informed evidence to guide inhibitor optimization and the design of combination therapies. Integrative multi-omics analyses coupled with artificial intelligence facilitate in-depth elucidation of complex resistance mechanisms and phenotypic transformation networks, promoting the identification of potential therapeutic targets. Systematized and algorithm-driven clinical decision support frameworks allow early prediction of resistance risk and dynamic optimization of treatment regimens, supporting the rational design of individualized therapies. As a platform integrating multidimensional molecular information, precision medicine-guided strategies establish both a theoretical foundation and a practical clinical framework for the dynamic management and sustained control of ALK-positive malignancies.

## 7. Future Perspectives

With the rapid advancement of high-throughput sequencing and precision molecular diagnostics [91], the identification of rare ALK fusion events across multiple solid tumors has continued to rise, broadening the landscape of ALK-driven malignancies. Looking forward, several areas are likely to shape the next phase of ALK-targeted therapy. First, the management of increasingly prevalent compound mutations and polyclonal resistance will require inhibitors with improved molecular selectivity and more rational sequencing strategies. This is particularly important for lesions involving the central nervous system, where enhanced blood–brain barrier penetration remains essential. Second, the integration of ctDNA profiling, multi-omics analysis, and AI-driven computational modeling is expected to transition from exploratory research to routine clinical decision-making, enabling real-time resistance monitoring and personalized therapeutic adjustment. Third, emerging modalities, such as targeted protein degraders and bispecific constructs, may provide alternative mechanisms capable of addressing resistance profiles beyond the reach of conventional TKIs. However, significant real-world implementation challenges remain. Limited access to repeated biopsies, high costs of next-generation sequencing, toxicity concerns associated with combination regimens, and regulatory hurdles may constrain the clinical translation of these innovations. A comprehensive understanding of these barriers will be critical to ensure that future ALK-directed strategies are both scientifically sound and practically feasible in clinical practice.

## 8. Conclusions

Over the past decade, ALK-targeted therapy has achieved notable clinical success, particularly in ALK-positive NSCLC and related malignancies. Sequential development from first- to third-generation inhibitors has effectively addressed key resistance mutations and improved central nervous system drug penetration, leading to substantial gains in progression-free and overall survival. Nonetheless, the heterogeneity and dynamic evolution of tumor resistance remain major challenges to long-term efficacy. Emerging multi-mechanistic combination strategies, coupled with precision medicine approaches including high-throughput omics and real-time molecular monitoring, offer opportunities for adaptive, individualized treatment optimization. Integration of AI-driven multi-omics analyses with mechanistic insights is poised to further enhance the precision, efficacy, and personalization of ALK-targeted therapy, providing a robust framework for improving clinical outcomes and informing future research directions.

## Data Availability

No new data were created or analyzed in this study. Data sharing is not applicable to this article.

## Abbreviations

The abbreviations used throughout this manuscript are listed below:

ALK	Anaplastic lymphoma kinase
RTK	Receptor tyrosine kinase
PTK	Protein tyrosine kinase
EMT	Epithelial–mesenchymal transition
NSCLC	Non-small cell lung cancer
SCLC	Small cell lung cancer
ALCL	Anaplastic large cell lymphoma
IMTs	Inflammatory myofibroblastic tumors
CNS	Central nervous system
ORR	Objective response rate
PFS	Progression-free survival
HDAC	Histone deacetylase
